# CGRP as a potential mediator for the sexually dimorphic responses to traumatic brain injury

**DOI:** 10.1186/s13293-024-00619-x

**Published:** 2024-05-30

**Authors:** Chunyan Li, Erum Ajmal, Khaled Alok, Keren Powell, Steven Wadolowski, Willians Tambo, Justin Turpin, Ernest Barthélemy, Yousef Al-Abed, David LeDoux

**Affiliations:** 1 Translational Brain Research Laboratory, The Feinstein Institutes for Medical Research, 350 Community Dr, Manhasset, NY 11030 USA; 2 Department of Neurosurgery, North Shore University Hospital, Manhasset, NY 11030 USA; 3 Elmezzi Graduate School of Molecular Medicine at Northwell Health, Manhasset, NY 11030 USA; 4 Division of Neurosurgery, SUNY Downstate College of Medicine, Brooklyn, NY 11203 USA; 5 Institute of Bioelectronic Medicine, The Feinstein Institutes for Medical Research, Manhasset, NY 11030 USA

**Keywords:** Traumatic brain injury, Calcitonin gene-related peptide, Sex hormones, Sexual dimorphism, Oxidative stress, Microvascular dysfunction, Memory, Anxiety, Depression

## Abstract

**Background:**

The outcomes of traumatic brain injury (TBI) exhibit variance contingent upon biological sex. Although female sex hormones exert neuroprotective effects, the administration of estrogen and progesterone has not yielded conclusive results. Hence, it is conceivable that additional mediators, distinct from female sex hormones, merit consideration due to their potential differential impact on TBI outcomes. Calcitonin gene-related peptide (CGRP) exhibits sexually dimorphic expression and demonstrates neuroprotective effects in acute brain injuries. In this study, we aimed to examine sex-based variations in TBI structural and functional outcomes with respect to CGRP expression.

**Methods:**

Male and female Sprague Dawley rats were exposed to controlled cortical impact to induce severe TBI, followed by interventions with and without CGRP inhibition. In the acute phase of TBI, the study centered on elucidating the influence of CGRP on oxidative stress, nuclear factor erythroid 2-related factor 2 (Nrf2) and endothelial nitric oxide synthase (eNOS) signaling in the peri-impact tissue. Subsequently, during the chronic phase of TBI, the investigation expanded to evaluate CGRP expression in relation to lesion volume, microvascular dysfunction, and white matter injury, as well as working and spatial memory, anxiety-like, and depression-like behaviors in subjects of both sexes.

**Results:**

Female rats exhibited elevated levels of CGRP in the peri-impact brain tissue during both baseline conditions and in the acute and chronic phases of TBI, in comparison to age-matched male counterparts. Enhanced CGRP levels in specific brain sub-regions among female rats correlated with superior structural and functional outcomes following TBI compared to their male counterparts. CGRP inhibition induced heightened oxidative stress and a reduction in the expression of Nrf2 and eNOS in both male and female rats, with the observed alteration being more pronounced in females than in males.

**Conclusions:**

This study marks the inaugural identification of CGRP as a downstream mediator contributing to the sexually dimorphic response observed in TBI outcomes.

## Highlights

Sexual dimorphism is evident in the levels of CGRP in peri-impact cerebral tissue both prior to and following TBI. Female rats demonstrate elevated and more pronounced sensitivity to CGRP expression in the context of TBI when contrasted with their male counterparts.

Increased levels of CGRP in female rats initiated Nrf2 and eNOS signaling during the acute stage of TBI, resulting in a reduction of lesion volume by alleviating microvascular, white matter, and hippocampal injury in the chronic phase of TBI, as compared to male rats.

Elevated CGRP levels within specific brain sub-regions were associated with enhanced working and spatial memory function, as well as reduced anxiety- and depression-like behaviors in female rats compared to their male counterparts following TBI.

The roles of CGRP levels vary across different degrees of tissue injury severity, ranging from deleterious to potentially beneficial, necessitating further exploration and investigation.

## Plain language summary

Investigating sex disparities in traumatic brain injury (TBI) is crucial for the advancement of precision therapeutics. Despite the neuroprotective effects demonstrated by female sex hormones, the administration of estrogen and progesterone has not produced conclusive results. Therefore, it is conceivable that additional mediators, separate from female sex hormones, warrant consideration due to their potential differential influence on TBI outcomes. In this study, we examined sex-related variations in calcitonin gene-related peptide (CGRP) expression in peri-impact brain tissue and investigated its potential implications on associated TBI outcomes. CGRP exhibits sexually dimorphic expression and exerts a multifaceted influence on diverse physiological processes that contribute to the pathophysiology of TBI. Our findings reveal that female rats exhibit heightened CGRP levels at both baseline and post-TBI within specific brain sub-regions, thereby contributing to superior structural and functional outcomes compared to their age-matched male counterparts. Additionally, we identified substantial sex-based variations in mechanisms modulated by CGRP pertaining to oxidative stress and microvascular dysfunction. The disparities in CGRP levels may be crucial for comprehending the advantageous outcomes noted in female TBI. Therefore, elucidating the sex-related distinctions in CGRP within TBI brains could pave the way for improved management and treatment strategies for TBI in both male and female individuals.

## Background

Traumatic brain injury (TBI) outcomes exhibit variability influenced by biological sex [1–3]. The sexually dimorphic variation in TBI severity diminishes with aging in females, concurrent with a decline in female sex hormone levels [4–6]. Despite previous findings demonstrating the neuroprotective effects of female sex hormones, the administration of estrogen and progesterone has not yielded conclusive outcomes [4, 7, 8]. Some phase II clinical trials resulted in favorable outcomes [9, 10], namely a diminution in mortality and better neurological outcomes on 6 months follow up post-TBI. Other studies, however, reported no significant changes [11, 12]. This could be attributed to challenges in adaptive design to clinical trials or treatment regimen. However, it is also plausible that female sex hormones may not be the primary mediators of the sexually dimorphic response in TBI outcomes, or that there exist other downstream mediators within the female sex hormone pathway [4, 13]. Hence, it would be valuable to examine the effects of downstream mediators regulated by female hormones.

One potential sex-differentiated downstream mediator is calcitonin gene-related peptide (CGRP), the expression of which is partially modulated by progesterone and estrogen [14–20] and has shown protective effects in acute brain injuries including TBI, inducing vasodilation, anti-inflammation, and anti-oxidation [21–28]. CGRP is a 37-amino acid neuropeptide and has shown sex differences in expression, regulation, function, and behavior in both pre-clinical animal studies and human pain studies [17–20, 29–32]. Past research has indicated that female sex hormones have the capacity to initiate the activation of the trigeminovascular system, leading to the subsequent release of CGRP [33–38]. Furthermore, these hormones exert their influence on sensory neurons in both the central and peripheral nervous systems, thereby playing a contributory role in CGRP release [39, 40]. Females exhibit naturally higher baseline levels of CGRP and its receptor subunits [14, 35, 41–46], both of which decrease in the plasma and resistance arteries with aging [17, 47, 48], directly correlating with a concurrent decrease in female sex hormone levels [17, 48]. These investigations have demonstrated the association between female sex hormones and CGRP; nevertheless, the potential contribution of CGRP to sexually dimorphic responses in TBI outcomes remains a subject of uncertainty.

Thus, in the present study, we explored the influence of CGRP levels on sexually dimorphic responses to both acute and chronic traumatic brain injury by utilizing age-matched male and female rats, both with and without CGRP inhibition. Our results demonstrate sex differences in lesion volume development, oxidative stress, microvascular dysfunction, white matter and hippocampal injury, memory, anxiety-like and depression-like outcomes depend on CGRP levels at corresponding brain sub-regions.

## Materials and methods

### Animals

A total of 66 age-matched male and female rats (Sprague-Dawley, 9–10 weeks old, Charles River Laboratories, New York, USA) were used. Of these rats, 12 males and 12 females underwent acute TBI testing, and 9 males and 9 females underwent chronic TBI testing. For acute TBI, 12 male or female rats were further divided into 6 with CGRP inhibition and 6 without CGRP inhibition. A total of 12 males and 12 females were allocated to the sham-operated groups. Animals were housed in a temperature- and humidity-controlled room under a reverse 12:12 light: dark cycle. All experimental procedures were approved by the Institutional Animal Care and Use Committee of the Feinstein Institutes for Medical Research and performed in accordance with the National Institutes of Health Guidelines for the Use of Experimental Animals.

### Rat traumatic brain injury model

The controlled cortical impact (CCI) model was utilized to induce TBI in Sprague-Dawley rats using previously described methods [49, 50]. Briefly, animals were anesthetized with 4% isoflurane in medical air and were placed in a supine position with the head fixed in a stereotaxic frame. During the procedure, anesthetic levels were maintained at 1.5% isoflurane in medical air and body temperature at 36.5 ± 0.2 °C. A 6-mm craniotomy was performed over the parietal cortex, at halfway between lambda and bregma. CCI was delivered at the craniotomized portion of the skull using an electromagnetic-based device (Impact OneTM Stereotaxic CCI Instrument, Leica Biosystems) using previously established parameters (5 mm impactor diameter; 6 m/s velocity; 3 mm penetration; 100 ms dwell time) [51]. Sham rats underwent the same incision and craniotomy, without the induction of CCI. Following TBI induction or sham surgery, the incisions were sutured with 4 − 0 silk suture, and animals were treated with buprenorphine and topical antibiotics, prior to being placed in a clean cage for observation. Animals were provided with wet food and hydrogel (ClearH_2_O, USA) for the first 3-days following CCI, as well as extra enrichment for the duration of the survival study. Daily monitoring was in place for animals within the extended survival groups. No deaths occurred as a consequence of the CCI itself and animals were observed to return to normal cage exploratory activity within 24-hours following CCI.

### Experimental groups

**Fig. 1:**
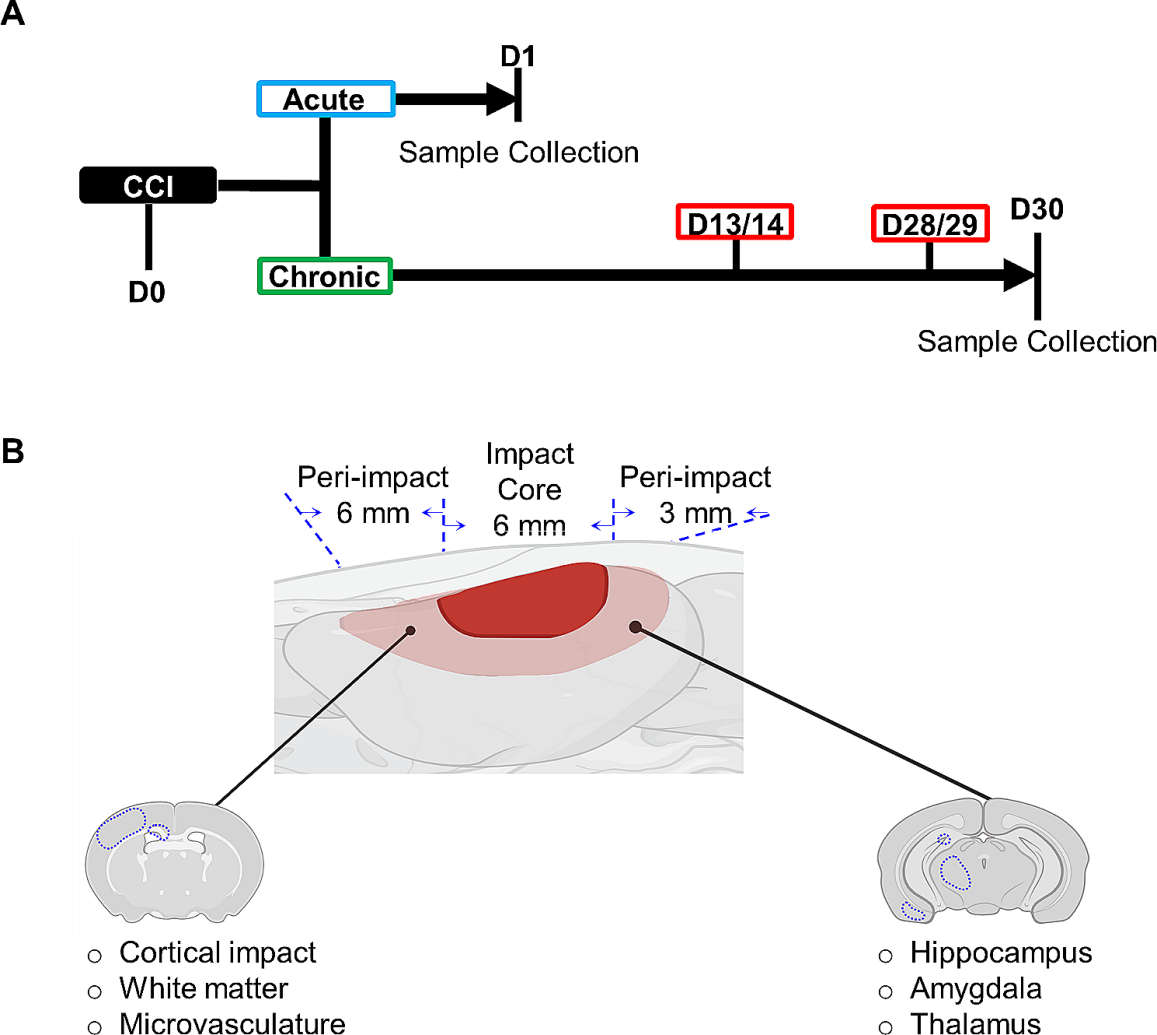
**A** Timeline of the experimental procedures. Controlled cortical impact (CCI) was induced on D0, at the same time as CGRP_8-37_ administration, for animals in CGRP_8-37_ group. Animals in the acute group were collected on D1, while animals in the chronic group were collected on D30 (red boxes indicate time of behavior assessment). **B** Definition of impact core and peri-impact brain tissue for analysis

66 age-matched male and female rats of were divided into the following 5 experimental groups as shown in Fig. 1A: (1) male/female sham group for acute TBI (24 h after sham surgery); (2) male/female sham group for chronic TBI (30 d after sham surgery); (3) male/female vehicle group for acute TBI (24 h after CCI); (4) male/female vehicle group for acute TBI with CGRP inhibition (24 h after CCI); (5) male/female vehicle group for chronic TBI (30 d after CCI). CGRP_8 − 37_ (Tocris Bioscience, USA), a peptide antagonist for CGRP receptors, was injected via the femoral vein cannula at 200 µg/kg, immediately after CCI-induced TBI for CGRP inhibition groups [52–54]. Group sample sizes were planned based on a power of 0.800, a statistical significance of 0.05, and a hypothesized standard deviation of 15% based on previous experimental results. Sample sizes for chronic behavioral assessments were increased intentionally to account for a higher degree of inter-animal variability, based on prior experimental observations. 6 male/female rats were assigned to the acute TBI groups for fresh collection, and 9 male/female rats were assigned to the chronic TBI groups for transcardial perfusion.

### Fresh tissue sample preparation and measurement

#### Peri-impact brain tissue collection

Peri-impact brain tissue was freshly collected at 24 h after CCI to measure markers of oxidative stress and microvascular dysfunction. Animals were heavily anesthetized using 5% isoflurane and then decapitated and the brains were removed. Anterior and posterior peri-impact tissues were collected using a brain matrix, as shown in Fig. 1B, and further divided into ipsilateral and contralateral tissue. The two ipsilateral peri-impact tissues were combined, powered in liquid nitrogen, and stored at -80 °C until analysis.

#### Biochemical assessment

Powdered ipsilateral peri-impact tissue samples were homogenized in radioimmunoprecipitation assay (RIPA) lysis buffer containing protease and phosphatase inhibitor cocktail (Thermo Fisher Scientific, USA). Following lysis, the homogenate was centrifuged for 5 min at 4 °C, supernatants were collected and total protein concentration was quantified using the BCA protein assay kit (Thermo Fisher Scientific, USA). Protein samples were separated on an SDS-polyacrylamide gel by electrophoresis, according to molecular weight. Proteins were then electro‐transferred onto polyvinylidene difluoride membranes using the semi-dry transfer method, blocked with 5% skimmed milk at room temperature for 1 h and then incubated with primary antibodies including anti-nitrotyrosine (1:1000, NT, mouse, Abcam, USA) [55, 56], anti-CGRP (1:1000, CGRP, mouse, Santa Cruz Biotechnology, USA), anti-phosphorylated Nrf2 (1:1000, pNrf2, rabbit, Abcam, USA), anti-endothelial nitric oxide synthase (1:1000, eNOS, rabbit, Thermo Fisher Scientific, USA) [57] overnight at 4 °C. After three washes in Tris‐buffered saline with Tween 20 (TBST), the membranes were incubated with secondary antibodies (Goat anti-Rabbit-HRP, Abcam, USA; Goat anti-Mouse-HRP, Abcam, USA) at room temperature for 1-hour. Signals were detected by chemiluminescence using ECL substrate (Thermo Fisher Scientific, USA) on a BioRad ChemiDoc Imaging System. ImageJ was used to quantify relative protein levels in the blots. β-actin (Sigma, USA) was used as a loading control for calculation purposes. The reduced glutathione level in peri-impact brain tissue was determined according to the manufacturer’s protocol using the GSH/GSSG Ratio Detection Assay Kit (Fluorometric-Green, Abcam, USA).

### Fixed brain tissue preparation for cryosectioning and staining

#### Brain perfusion, fixation and cryosectioning

At 30 days after CCI, animals were heavily anesthetized using 5% isoflurane and transcardially perfused with 0.01 M phosphate-buffered saline (PBS) followed by 4% paraformaldehyde (PFA) solution. The head was then removed via guillotine and the brain was collected. Samples were placed in 4% PFA overnight, followed by graded sucrose solutions, and cryo-embedding in a 3:1 mixture of Optimal Cutting Temperature compound (Electron Microscopy Sciences, USA) and 30% sucrose in PBS. Following embedding, samples were placed in -80 °C storage. Brains were coronally cryosectioned every 400 μm at 18 μm thickness from caudal to rostral, mounted on Superfrost Plus glass slides (Thermo Fisher Scientific, USA) and Polysine glass slides (Thermo Fisher Scientific, USA), and stored at -30 °C until staining.

#### Morphological assessment

To assess morphological damage, samples mounted on Superfrost Plus slides were stained with hematoxylin and eosin (H&E). For assessment of lesion volume, digital images of the slides were acquired using the Pathscan Enabler 5 (Meyer Instruments, USA). Lesion volume was calculated manually using the ImageJ tracing tool for each section. Section areas were summed and converted using the known pixel size to calculate total volume (mm^3^) for each animal.

For assessment of cellular damage in regions of interest (ROI), slides were imaged using EVOS M7000 (Thermo Fisher Scientific, USA). ROIs were identified using the Waxholm Space atlas for anatomic landmark verification and imaged at low power (10X = 0.80 mm^2^), medium power (20x = 0.2mm^2^) and high power (40x = 0.05mm^2^). Cellular health was quantified within the dentate gyrus (DG), amygdala and thalamus, and expressed as percentage of unhealthy neurons per ROI (medium power) using references of normal cellular formation within each structure as a guide.

As a measure of microvascular dysfunction, pial and parenchymal arterioles were measured. In brief, the thickness and inner diameter of 3 pial vessels from each brain were measured. To minimize the degree of variation due to cryosectioning-induced vessel deformation, measurements were taken along three points of each vessel and averaged. A ratio of vessel thickness to diameter was also calculated to account for the natural variation in size between pial vessels at baseline. For parenchymal arterioles, the number of constricted microvessels was quantified.

For immunofluorescent assessment of neuronal and white matter damage, and brain region specific CGRP expression, cryosectioned tissues on Polysine coated slides were incubated with primary antibody (NeuN, (1:500, mouse, Abcam, USA), CGRP (1:50, CGRP, mouse, Abcam, USA)) and their corresponding secondary antibody, and were counterstained with DAPI (1:2000, Thermo Fisher Scientific, USA) and mounted with Vectashield Antifade mounting medium (Vector Laboratories, USA). Slides were visualized and imaged with EVOS M7000 imaging system (Thermo Fisher Scientific, USA) using the 20x objective. Area of intact white matter was measured using the threshold function in ImageJ to isolate tissues expressing myelin basic protein (MBP) and quantify their total area. CGRP expression was measured using the ImageJ mean gray value function.

### Assessment of memory, anxiety-like and depression-like behavior

Behavioral assays established in prior studies for rats with subarachnoid hemorrhage were implemented in the current investigation [58]. Prior to TBI induction, male/female rats in chronic TBI groups were assessed for baseline memory and neuropsychological function. Animals underwent TBI induction on day zero, and then underwent working and long-term spatial memory assessment (Y maze), anxiety-like assessment (Elevated plus maze) and depression-like assessment (Porsolt forced swim) on day 14 and day 28 (Fig. 1A). All assessments were performed in a dedicated suite, with minimal noise or external stimuli and consistent lighting. Assessments were performed at the same time each day, in order of least stressful to most stressful, with time in between assessments for the animals to relax. Animal activity was tracked using the Ethovision software (Version XT 16, Noldus Information Technology, Netherlands), and verified by an experienced technician to ensure accuracy. Between each animal, surfaces and apparatuses were cleaned with Peroxiguard solution followed by 75% ethanol. Animals were euthanized at 30 days following CCI.

#### Working and long-term spatial memory assessment

The Y-Maze test is comprised of a Y-shaped structure composed of three tunnels joined at 120° angles and can be used to assess different aspects of memory, including both working and long-term spatial memory. The percentage of complete alternations, defined as a rat visiting all three arms in a row with no repeats, is used to approximate the degree of spatial working memory dysfunction. The long-term spatial memory assessment relies on exposing the rats to the maze for a 10-min pre-training period with one arm closed off. After a 4-hour interval, the rats are again placed into the structure with all three arms open for a 5-min period. The number of entrances to the novel arm is used as a measure of long-term spatial memory dysfunction.

#### Anxiety-like behavior assessment

To assess anxiety-like behavior, rats underwent the elevated plus maze assessment. The maze consists of four arms set perpendicularly to one another to form a cross. Two of the arms are open to the outside and two of the arms are surrounded by opaque walls on three sides, to form a semi-enclosed space. Each rat is placed in the center of the maze facing towards an open arm and away from the assessor. The rat is allowed 5 min to explore the maze freely; the number of entries into each arm and the amount of time spent in each arm is recorded. This is represented as percentage of total entries/time. If a rat freezes or falls from the maze, it is disqualified from the test.

#### Depression-like behavior assessment

To assess depression-like behavior, rats underwent the Porsolt Forced Swim test. Rats are placed into an acrylic cylinder (50 cm x 20 cm) filled with water within which they cannot touch the bottom and from which they cannot climb out. The degree to which they cease struggling is taken as a measure of situational despair and, as such, an analogue for depression. In this case, an animal’s immobility is classed as the lack of movement except for that which is necessary to keep their noses above the water.

### Statistical analyses

All data are expressed as mean ± standard deviation and the statistical analyses were performed using GraphPad Prism software (GraphPad Software 9.0.3). Grubb’s test was used to identify any statistical outliers and normal distributions of the data was confirmed with Shapiro-Wilk test. Differences between two groups were determined using unpaired Student t-tests (two tailed) when normally distributed or the non-parametric Mann-Whitney U tests when not. Differences between more than two groups were determined using one-way ANOVA followed by a Tukey post hoc test. *P* values of less than 0.05 were considered statistically significant.

## Results

### Sex differences in lesion volume development correlate with CGRP levels at the peri-impact cortex

**Fig. 2:**
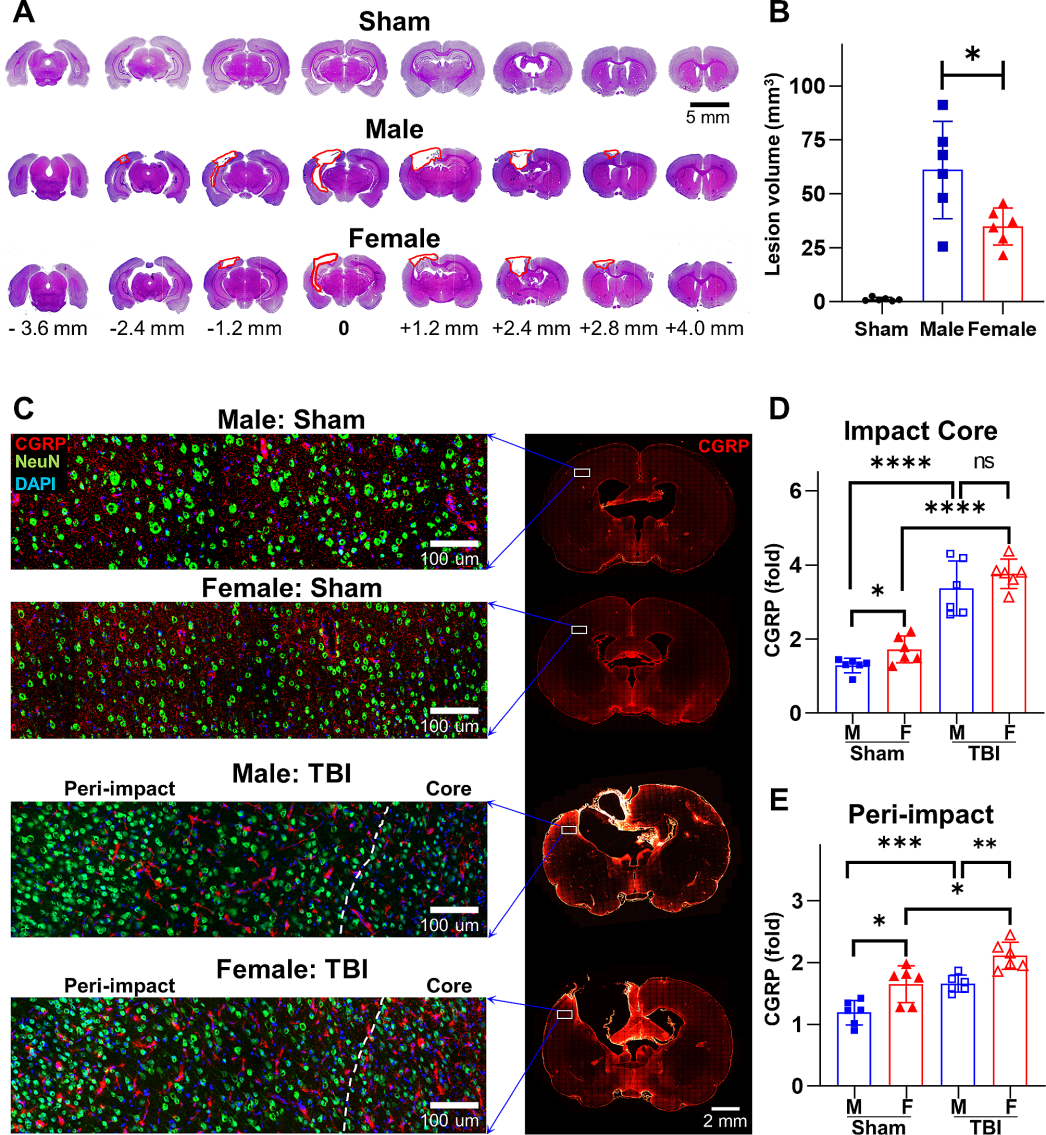
Sex differences in lesion volume development correlate with CGRP levels at the peri-impact cortex. **A** Representative H&E stained images for lesion volume. Due to variations in lesional development between males and females, “0” indicates lesion center, rather than bregma. **B** Quantified lesion volume at 30 d after CCI. **C** Representative immunofluorescent stained images for CGRP and NeuN expression at the impact core and peri-impact brain tissue. **D** Quantified CGRP levels at the impact core tissue. **E** Quantified CGRP levels at the peri-impact tissue. *p < 0.05, **p < 0.01, ***p < 0.001, ****p < 0.0001

At 30 d following TBI, both male and female rats experienced significant lesion development compared to sham rats (Fig. 2A). Compared to their age-matched male counterparts, females exhibited more than 44.5% less lesion volume development (TBI-male: 62.66 ± 19.67 mm^3^, TBI-female: 34.79 ± 8.58 mm^3^, *p* = 0.0242; Fig. 2B).

The expression of CGRP in the brain was assessed by immunofluorescence (Fig. 2C). In sham brains, female rats exhibited elevated CGRP expression in various regions of the cortex compared to their male counterparts (Impact core region: sham-male: 1.28 ± 0.19, sham-female: 1.72 ± 0.36, *p* = 0.026; Peri-impact region: sham-male: 1.18 ± 0.19, sham-female: 1.64 ± 0.29, *p* = 0.010; Fig. 2D). At 30 d following TBI, CGRP levels exhibited a significant increase in the impact core regions compared to sham brains, with no discernible statistical difference observed between male and female rats (Impact core region: TBI-male: 3.37 ± 0.74, TBI-female: 3.76 ± 0.39, *p* = 0.276; Fig. 2D). At the peri-impact region, however, female rats exhibited markedly elevated CGRP expression compared to their male counterparts (Peri-impact region: TBI-male: 1.66 ± 0.14, TBI-female: 2.11 ± 0.22, *p* = 0.0016; Fig. 2E).

### Sex differences in oxidative stress depend on CGRP levels in TBI brains

**Fig. 3:**
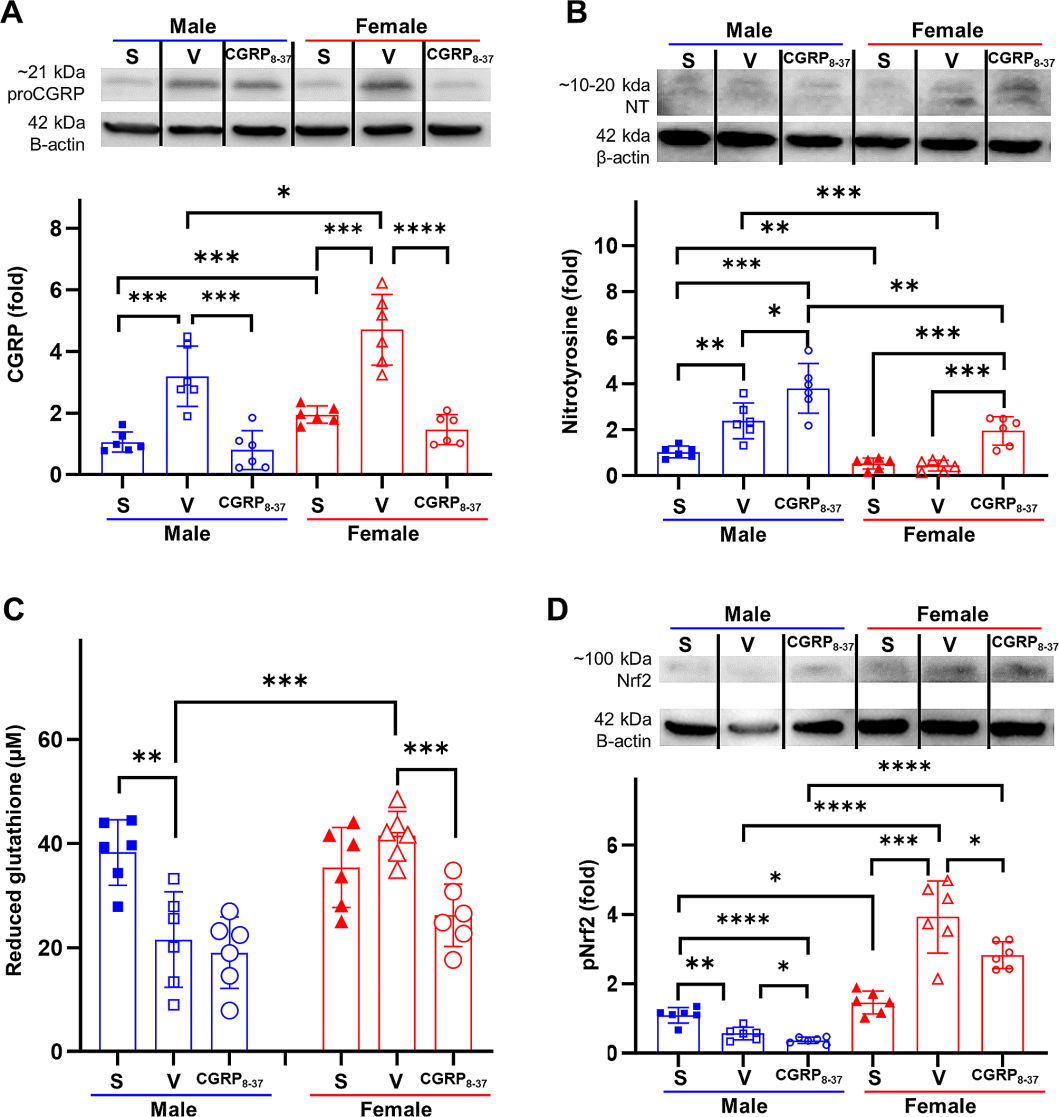
Sex differences in oxidative stress depend on CGRP levels. **A** Quantified CGRP levels after TBI with and without CGRP inhibition. **B** Quantified nitrotyrosine levels after TBI with and without CGRP inhibition. **C** Quantified reduced glutathione levels after TBI with and without CGRP inhibition. **D** Quantified pNrf2 levels after TBI with and without CGRP inhibition. *p < 0.05, **p < 0.01, ***p < 0.001, ****p < 0.0001

CGRP_8 − 37_, a peptide antagonist for CGRP receptors, was delivered immediately after CCI-induced TBI to investigate the contribution of CGRP in the sexually dimorphic responses to TBI. As shown in Fig. 3A, CGRP levels in the peri-impact tissue of the brain significantly increased in both male and female rats following TBI, with females demonstrating markedly higher levels (TBI-male: 3.19 ± 0.98, TBI-female: 4.70 ± 1.15, *p* = 0.0343). Following CGRP inhibition, there was a significant reduction in CGRP levels, with female rats displaying a more pronounced decrease compared to male rats in relation to their baseline levels (TBI-male-CGRP_8 − 37_: 0.76 ± 0.70 (vs. TBI-male *p* = 0.0005); TBI-female CGRP_8 − 37_: 1.46 ± 0.48 (vs. TBI-female *p* < 0.0001)).

One of the primary contributors to the severity of TBI in the acute timeframe is oxidative stress [59, 60]. Nitrotyrosine levels exhibited sex-dependent variations at 24 h following TBI, with a significant increase observed in male rats, indicating increased oxidative stress. In contrast, there was no discernible difference in female rats when compared to their respective baseline levels (Sham-male: 1.03 ± 0.26, TBI-male: 2.39 ± 0.77, *p* = 0.0022; Sham-female: 0.53 ± 0.24, TBI-female: 0.44 ± 0.23, *p* = 0.516; Fig. 3B). This aligns with the pattern of elevated baseline CGRP levels and the subsequent further increase in CGRP observed in female rats following TBI, surpassing that of males. CGRP inhibition led to a notable augmentation in nitrotyrosine expression in both male and female rats compared to TBI vehicle rats, with female rats exhibiting more pronounced exacerbation in oxidative stress (TBI-male-CGRP_8 − 37_: 3.79 ± 1.08, TBI-female CGRP_8 − 37_: 1.94 ± 0.62, *p* = 0.0688).

Assessment of reduced glutathione levels indicated a similar, though not identical, pattern (Fig. 3C). At baseline, males and females expressed similar levels of glutathione (Sham-male: 38.29 ± 6.29, Sham-female: 35.42 ± 7.70, *p* = 0.4954). Following severe TBI, however, females exhibited an elevation in glutathione levels, whereas males demonstrated a notable decrease in glutathione levels (TBI-male: 21.57 ± 9.17, TBI-female: 41.54 ± 4.67, *p* = 0.0008). CGRP inhibition led to marked reductions in glutathione levels in both males and females, with females exhibiting a more pronounced decrease compared to their own TBI-vehicle levels (TBI-male-CGRP_8 − 37_: 19.04 ± 6.86, TBI-female CGRP_8 − 37_: 26.24 ± 6.04).

Nrf2 is a master regulator of oxidative stress and inflammation following TBI [61–63], which has been previously shown to be upregulated by CGRP [64]. Phosphorylated Nrf2 (pNrf2) levels at the peri-impact brain tissue were assessed at 24 h after TBI. Following TBI, the expression of pNrf2 increased in females, whereas it decreased in males (Sham-male: 1.09 ± 0.22, TBI-male: 0.56 ± 0.18, *p* = 0.0012; Sham-female: 1.46 ± 0.32, TBI-female: 3.93 ± 1.04, *p* = 0.0002; Fig. 3D). CGRP inhibition led to a reduction in the expression of pNrf2 in both male and female rats (TBI-male-CGRP_8 − 37_: 0.37 ± 0.09, TBI-female CGRP_8 − 37_: 2.83 ± 0.38).

### Sex differences in microvascular dysfunction depend on CGRP levels in TBI brains

**Fig. 4:**
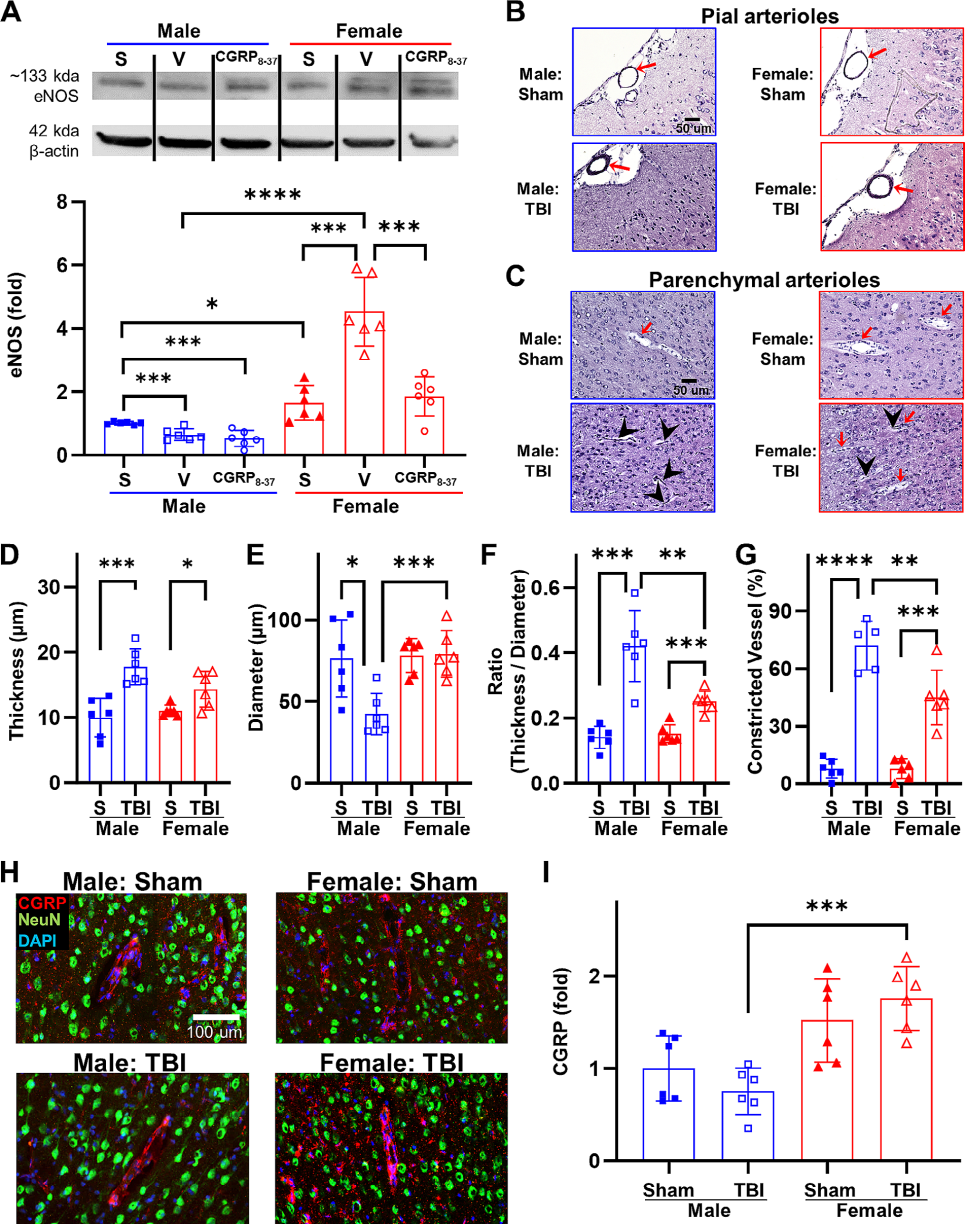
Sex differences in microvascular dysfunction depend on CGRP levels. **A** Quantified eNOS levels after TBI with and without CGRP inhibition. **B** Representative H&E stained images for pial arterioles at 30 d after CCI. Red arrows indicate pial arterioles. **C** Representative H&E stained images for parenchymal arterioles at 30 d after CCI. Red arrows indicate opened parenchymal arterioles. Black arrow heads indicate constricted parenchymal arterioles. **D** Quantified wall thickness of pial arterioles. **E** Quantified vessel diameter of pial arterioles. **F** Quantified wall thickness to vessel diameter ratio of pial arterioles. **G** Quantified constricted vessels of parenchymal arterioles. **C** Representative immunofluorescent stained images for CGRP surrounding parenchymal arterioles. **I** Quantified CGRP levels around the parenchymal vessels after 30 d TBI. *p < 0.05, **p < 0.01, ***p < 0.001, ****p < 0.0001

The disruption of microvessels significantly contributes to the progression of chronic damage and impairment following TBI [65]. In the absence of injury, female rats exhibited a higher baseline level of eNOS expression compared to male rats (Sham-male: 1.02 ± 0.05, Sham-female: 1.65 ± 0.54, *p* = 0.0168; Fig. 4A). At 24 h post-TBI, the assessment of eNOS expression in the peri-impact brain tissue indicated elevated levels in female rats and reduced levels in male rats (TBI-male: 0.65 ± 0.18, TBI-female: 4.53 ± 1.08, *p* < 0.0001; Fig. 4A).

At 30 d after CCI, pial and parenchymal arterioles were assessed as a measure of microvascular disruption. As shown in Fig. 4B and C, following TBI, there were significant alterations observed in both the vessel wall thickness and diameter of pial and parenchymal arterioles in both male and female rats. In the case of pial arterioles, female rats exhibited a lesser wall thickness compared to male rats, albeit without reaching statistical significance (Sham-male: 10.02 ± 2.95, Sham-female: 11.04 ± 0.87, *p* = 0.4331; TBI-male: 17.79 ± 2.76, TBI-female: 14.34 ± 2.72, *p* = 0.0539; Fig. 4D). Moreover, female rats demonstrated a larger diameter of pial arterioles compared to male rats (Sham-male: 76.39 ± 23.53, Sham-female: 78.15 ± 10.43, *p* = 0.8700; TBI-male: 42.33 ± 12.62, TBI-female: 79.17 ± 14.27, *p* = 0.0008; Fig. 4E). This trend was preserved when correcting for baseline pial vessel size variation with a thickness/diameter ratio (Sham-male: 0.14 ± 0.03, Sham-female: 0.15 ± 0.03, *p* = 0.5631; TBI-male: 0.42 ± 0.11, TBI-female: 0.25 ± 0.03, *p* = 0.0045; Fig. 4F). For parenchymal arterioles, female rats showed much less constricted vessels (Sham-male: 7.67 ± 5.00, Sham-female: 7.63 ± 5.15, *p* = 0.9889; TBI-male: 71.99 ± 12.64, TBI-female: 44.93 ± 14.19, *p* = 0.0092; Fig. 4G). Such enhancement in microvascular function in female rats was associated with elevated CGRP expression surrounding parenchymal arterioles shown in Fig. 4H. In comparison to male rats, female rats demonstrated elevated CGRP expression both before and after TBI (Sham-male: 1.00 ± 0.47, Sham-female: 1.58 ± 0.33, *p* = 0.0334; TBI-male: 1.43 ± 0.38, TBI-female: 1.92 ± 0.34, *p* = 0.0403; Fig. 4G).

### Sex differences in white matter and hippocampal injury correlate with CGRP levels in TBI brains

**Fig. 5:**
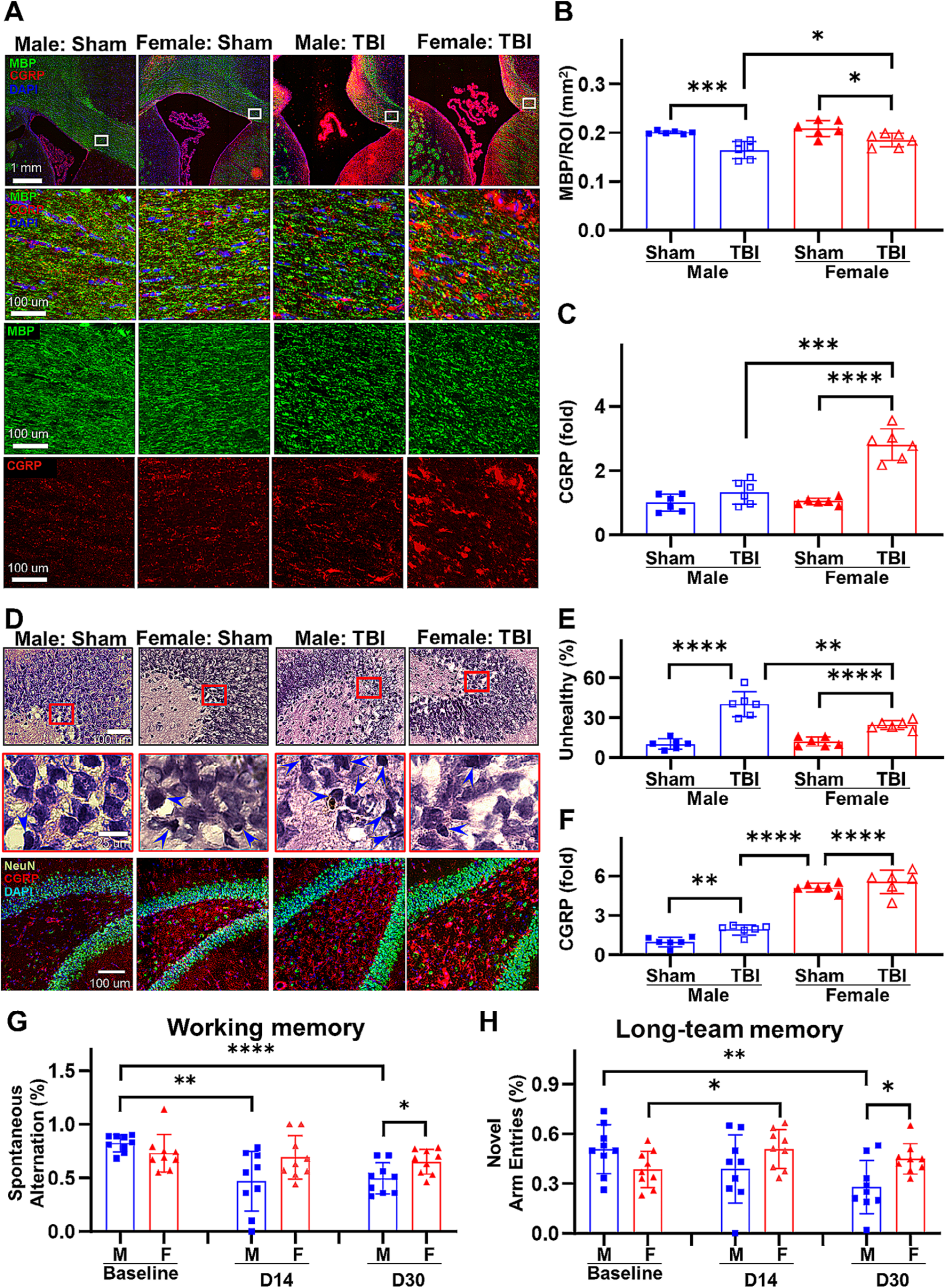
Sex differences in white matter and hippocampal injury depend on CGRP levels. **A** Representative immunofluorescent stained images for CGRP and MBP at the corpus callosum. **B** Quantified MBP levels at 30 d after TBI. **C** Quantified CGRP levels in the corpus callosum at 30 d after TBI. **D** Representative H&E stained and immunofluorescent stained images for CGRP and NeuN expression at dental gyrus of hippocampus. Blue arrow heads indicate injured cells. **E** Quantified unhealthy cell counts at dental gyrus. **F** Quantified CGRP levels at dental gyrus. **G** Quantified spontaneous alternation for working spatial memory assessment. **H** Quantified novel arm entrance for long-term spatial memory assessment. *p < 0.05, **p < 0.01, ***p < 0.001, ****p < 0.0001

Female rats displayed a significantly greater preservation of intact white matter following TBI, as evidenced by MBP immunohistochemical staining, in comparison to their age-matched male counterparts shown in Fig. 5A. In the corpus callosum, female rats exhibited markedly elevated MBP signaling 30 d post-TBI (Sham-male: 0.20 ± 0.00 mm^2^, Sham-female: 0.21 ± 0.02 mm^2^, *p* = 0.2609; TBI-male: 0.16 ± 0.02 mm^2^, TBI-female: 0.19 ± 0.01 mm^2^, *p* = 0.0500; Fig. 5B). The protection of white matter in female rats following TBI is associated with the elevated level of CGRP (Sham-male: 1 ± 0.26, Sham-female: 1.05 ± 0.10, *p* = 0.6966; TBI-male: 1.33 ± 0.37, TBI-female: 2.81 ± 0.49, *p* = 0.0001; Fig. 5C).

Furthermore, female rats exhibited reduced hippocampal damage characterized by a lower quantity of unhealthy cells, as indicated by hematoxylin and eosin (H&E) staining (Fig. 5D). In dental gyrus (DG), female rats showed much less unhealthy neurons than male rats (Sham-male: 10.35 ± 3.85, Sham-female: 12.17 ± 3.31, *p* = 0.4005; TBI-male: 40.33 ± 9.49, TBI-female: 24.61 ± 3.28, *p* = 0.0033; Fig. 5E). The enhancement in cellular health within the hippocampal subfield is strongly correlated with the heightened expression of CGRP in female rats (Sham-male: 1 ± 0.36, Sham-female: 5.12 ± 0.34, *p* < 0.0001; TBI-male: 1.91 ± 0.38, TBI-female: 5.55 ± 0.88, *p* < 0.0001; Fig. 5F).

Reduction in white matter and hippocampal injuries in female rats resulted in improved working and long-term spatial memories, as evaluated through Y-maze testing. There is no significant difference between baseline memories for male and female rats (Fig. 5G). However, at 30 d after CCI, male rats demonstrate pronounced dysfunction in both working and long-term spatial memories compared to their female counterparts (Fig. 5H).

### Sex differences in anxiety- and depression-like behavior correlate with CGRP levels in TBI brains

**Fig. 6:**
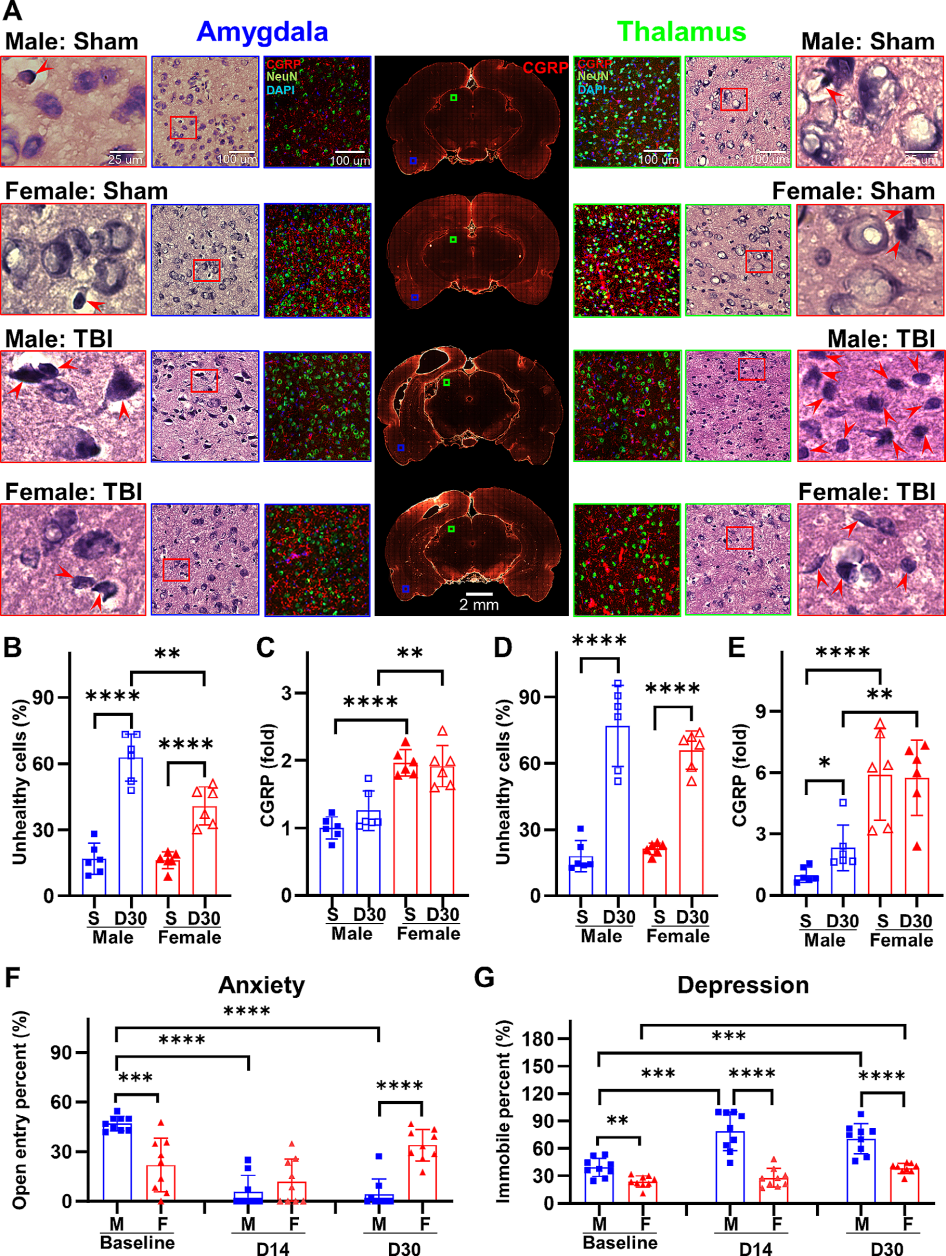
Sex differences in anxiety- and depression-like behavior depend on CGRP levels. **A** Representative H&E stained and immunofluorescent stained images for CGRP and NeuN at amygdala and thalamus. **B** Quantified unhealthy cell counts at amygdala at 30 d after TBI. **C** Quantified CGRP levels at amygdala at 30 d after TBI. **D** Quantified unhealthy cell counts at thalamus at 30 d after TBI. **E** Quantified CGRP levels at thalamus at 30 d after TBI. **F** Quantified open arm entry for anxiety-like behavior assessment. **G** Quantified immobile percent for depression-like behavior assessment. *p < 0.05, **p < 0.01, ***p < 0.001, ****p < 0.0001

Following CCI, brain regions beyond the cortex were also impacted. We conducted an analysis of cellular injury in the amygdala and thalamus, regions situated at a distance from the direct cortical impact (Fig. 6A). These brain regions regulate anxiety and depression, both of which are associated with CGRP signaling. In amygdala, at 30 d after CCI, female rats showed less cellular injury than male rats (Sham-male: 16.90 ± 6.98, Sham-female: 16.17 ± 3.87, *p* = 0.8260; TBI-male: 62.77 ± 10.68, TBI-female: 40.83 ± 8.58, *p* = 0.0029; Fig. 6B). The amelioration in injury is associated with the heightened expression of CGRP in female rats (Sham-male: 1.01 ± 0.17, Sham-female: 1.96 ± 0.19, *p* < 0.0001; TBI-male: 1.26 ± 0.29, TBI-female: 1.92 ± 0.31, *p* = 0.0035; Fig. 6C). The thalamus exhibited a similar trend in cellular health (Fig. 6D), and CGRP expression (Fig. 6E), as observed in the amygdala.

Sex-stratification was apparent in the assessment of chronic neuropsychological outcomes, encompassing symptoms akin to anxiety and depression. The elevated plus maze test revealed a significantly greater degree of anxiety in male animals with TBI compared to sham animals at both chronic time points (14 d and 28 d after CCI, Fig. 6F). This was evident from the reduced time spent in the open arms and fewer entries into the open arms, behaviors typically associated with higher anxiety levels. Female rats, on the other hand, demonstrated a diminishing level of anxiety relative to their baseline as TBI progressed. By day 28 post-TBI, females demonstrated significantly lower anxiety levels compared to their male counterparts. The Porsolt Forced Swim test was employed to evaluate contextual despair. By day 14 post-TBI, male rats allocated nearly 80% of the assessment period in an immobile state, signifying heightened levels of situational depression. This level of immobility persisted in male animals without a discernible decrease or a trend toward normalization. In contrast, female rats exhibited significantly lower levels of depression than their male counterparts (Fig. 6G).

## Discussion

**Fig. 7:**
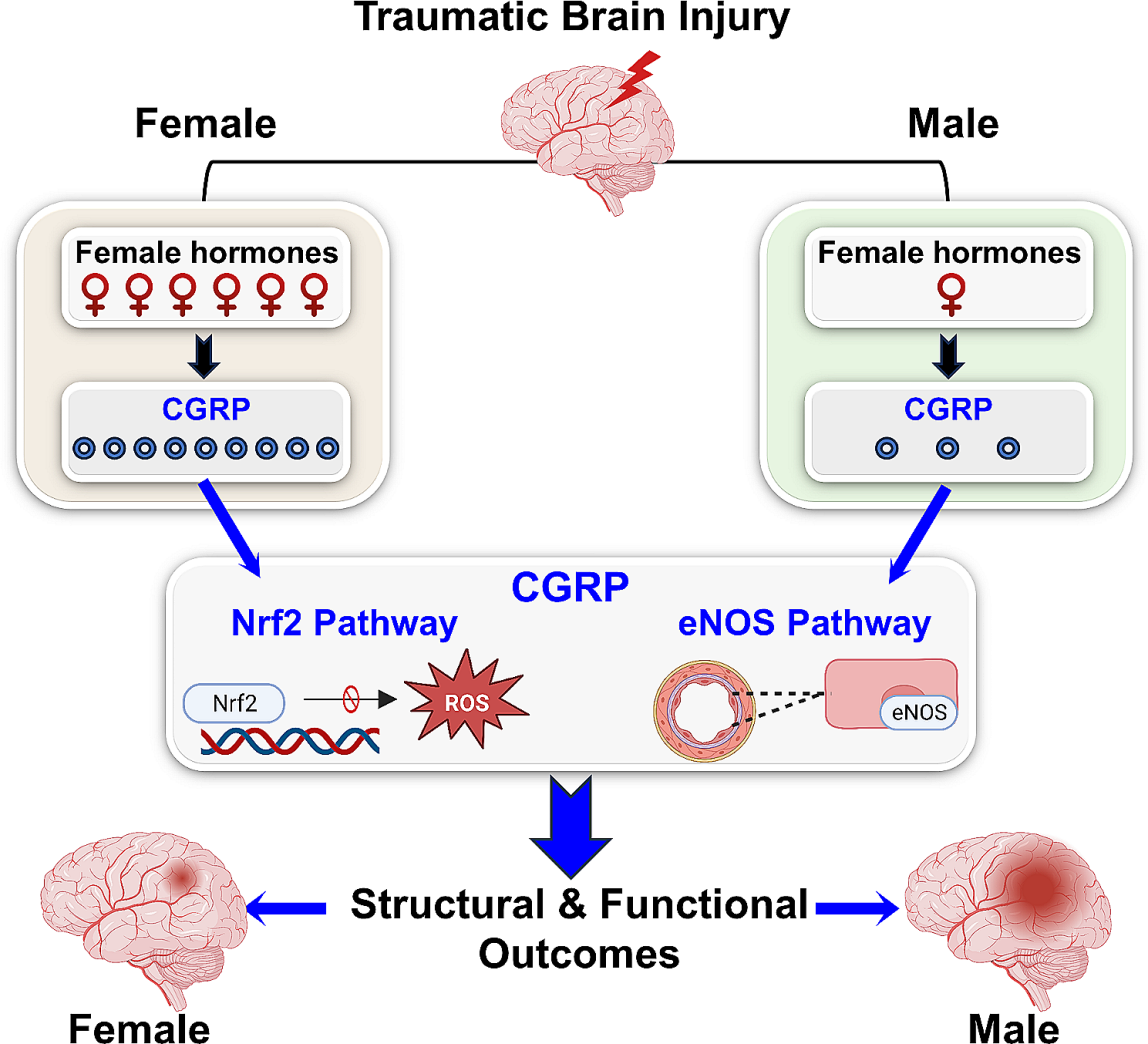
Conceptual diagram illustrating CGRP as a potential mediator for the sexually dimorphic responses to traumatic brain injury

In this study, we have demonstrated that CGRP is one of the downstream mediators behind the sexually dimorphic outcomes of TBI. Specifically, our investigation has identified a correlation between CGRP levels and the structural and functional outcomes of TBI. Elevated CGRP levels in females have been associated with superior outcomes compared to age-matched male counterparts, observed during both the acute and chronic phases of TBI. In comparison to CGRP levels in healthy tissue at baseline and injured tissue at the peri-impact and impact core, it is evident that there is a discernible therapeutic threshold, ranging from no effect to potentially beneficial or detrimental outcomes. Although the precise mechanisms by which female sex hormones modulate CGRP signaling in TBI brains, the various factors involved in such modulation, and the specific female sex hormone playing major roles in CGRP signaling remain elusive, our findings strongly indicate that elevated CGRP expression in female brains is associated with enhanced TBI outcomes, as elucidated in Fig. 7.

Sexual distinctions in CGRP levels were evident in various brain regions, including healthy, peri-impact, and impact core tissues, in the context of TBI. CGRP is recognized as a critical player in migraine pathophysiology [66]. In the context of injured brains, however, it has been demonstrated to confer neuroprotective effects by diminishing oxidative stress [64, 67], mitigating neuroinflammation [68], and modulating cerebral blood flow through vasodilation [69]. Prior investigations emphasize the significance of CGRP expression in correlation with female sex hormone concentrations in both normal and migraine-affected brains. For instance, evidence suggests that the extent of CGRP expression and CGRP axoplasmic transport changes with age in female rats, mirroring shifts in female sex hormones, which can be reversed by estrogen or progesterone application [70]. Estradiol and progesterone indirectly modulate CGRP synthesis in dorsal root ganglia, leading to an upregulation of CGRP mRNA both in vivo and in vitro [17, 71]. Notably, females exhibit priming to subthreshold CGRP in response to dural IL-6 or intracisternal brain-derived neurotrophic factor (BDNF) application, a phenomenon not observed in males [72]. However, as of now, there is no evidence indicating a connection between CGRP signals and sexually dimorphic responses in TBI outcomes. In this study, elevated CGRP levels were observed not only in healthy tissue, consistent with previous observations in intact female rats [21], but also in the peri-impact brain tissue of female rats, when compared to age-matched male rats. This observation extends across both the acute and chronic phases of TBI, where heightened CGRP levels correspond to improved outcomes in different brain sub-regions. Collectively, these pieces of evidence underscore the significance of regulating CGRP expression in conjunction with female sex hormone levels in the treatment of TBI.

Sexual dimorphism in CGRP levels has been noted regarding structural outcomes in TBI. Our results show that at the peri-impact brain tissue, higher CGRP levels observed in females correlated with better cellular health and structural integrity than males. Elevated CGRP levels in the peri-impact brain tissue, approximately 1.5-fold higher during the acute phase and 1.3-fold higher during the chronic phase of TBI, were associated with an ~ 47% reduction in lesion volume in female rats compared to age-matched male rats. Our findings are consistent with prior research indicating that intact female rats exhibit an ~ 50% reduction in lesional development compared to both male and ovariectomized female rats [6, 73]. Following severe TBI, we also observed varying degrees of impact in brain areas beyond the cortex, including the corpus callosum, hippocampus, amygdala, and thalamus. Higher levels of CGRP were detected in these brain sub-regions in females compared to males, and these elevated levels were associated with a reduction in unhealthy cells and preserved myelin integrity. Notably, within the impact core brain tissue, the levels of CGRP surpassed approximately 2-fold when compared to peri-impact cortical tissue in both sexes, lacking cellular improvement and exhibiting a noteworthy distinction between females and males. These findings imply the existence of a potential therapeutic threshold for CGRP in the injured brain, which varies with distinct levels of injury severity. Our results emphasize the crucial significance of modulating CGRP levels across diverse brain regions and at various stages of TBI severity.

While yet unexplored, the enhanced sex-dependent structural outcomes in TBI are hypothesized to emanate from the neuroprotective influences exerted by CGRP. Our study unveiled that following severe TBI, the pronounced disparity in oxidative stress between sexes was closely linked to the levels of CGRP in the brain. Female rats exhibited ~ 81% lower levels of nitrotyrosine than male rats after TBI. However, following the inhibition of CGRP, female rats displayed an approximately 3.4-fold exacerbation in nitrotyrosine levels, while male rats exhibited a 1.6-fold exacerbation compared to their respective TBI vehicle levels. It is noteworthy that reduced glutathione levels are correlated with CGRP levels, providing additional support for the role of CGRP as a crucial mediator in females for regulating oxidative stress levels. Our findings are consistent with prior research suggesting that CGRP regulates oxidative stress by inhibiting the production of reactive oxygen species ROS [74] and enhancing the expression of antioxidants [75–78]. Our investigation also revealed that elevated CGRP levels in female rats were associated with increased pNrf2 expression compared to males. As a redox-sensitive antioxidant gene regulating transcription factor, Nrf2 serves as a significant mediator of oxidative stress and inflammation in TBI, whereby its activation prevents oxidative stress, neuroinflammation, and neuronal apoptosis [63, 79]. At 24 h post-CCI, we noted a reduction in pNrf2 levels in male rats, whereas an increase was observed in female rats compared to their respective baselines. In male rats, inhibition of CGRP further lowered pNrf2 levels, while in female rats, CGRP inhibition resulted in decreased pNrf2 levels compared to the TBI-vehicle group, although these levels remained higher than baseline. These results emphasize the indispensable involvement of CGRP in the initiation of Nrf2 signaling, revealing responses that are contingent on the individual’s sex. Several preceding indications lend support to our findings. For instance, estradiol has been demonstrated to activate Nrf2 through the PI3K/AKT pathway [80, 81]. Moreover, existing literature indicates that CGRP also activates Nrf2 in glial cells through the activation of the PI3K/AKT pathway [49], as well as via the RAS/RAF/MEK pathway in smooth muscle cells [82]. While previous studies have elucidated individual associations between female sex hormones and Nrf2, as well as between CGRP and Nrf2, there is currently no exploration of the sex-dependent influence of CGRP on Nrf2 activation in TBI brains. Consequently, it holds promise for future investigations to explore the pathways linking female sex hormones to CGRP and Nrf2 in the context of TBI conditions.

The involvement of CGRP in vasodilation constitutes an additional element contributing to enhanced structural outcomes in TBI [14, 83–85]. Our observations suggest that CGRP levels influence sex-dependent eNOS expression both before and after TBI. Specifically, male rats demonstrated not only a lower baseline expression of eNOS but also a further decrease in its expression during the acute phase of TBI, correlating with aggravated microvascular impairment during the chronic phase. In contrast, females exhibited not only higher baseline expression but also increased expression following TBI, contributing to enhanced microvascular health during the chronic phase of TBI. Estrogen has been linked to increased activity of eNOS [86–88], along with the dilation of pial microvessels during ischemic conditions [89, 90]. eNOS assumes a crucial role in sustaining CBF following TBI and represents a pivotal component in the vasoactive properties associated with CGRP [78, 91]. Consequently, enhancing microcirculation after TBI through the modulation of CGRP expression may prove to be a significant factor in improving TBI outcomes in both sexes.

Our investigation may shed light on the role of CGRP signaling in the sex differences observed in functional outcomes following TBI. In the aftermath of TBI, a contributor to chronic morbidity is the occurrence of white matter and hippocampal injuries, which may endure for prolonged periods post-contusion and contribute to the onset of vascular dementia [92, 93]. Notably, we observed an enhanced degree of myelin confluence in the corpus callosum of female rats, aligning with elevated levels of acute and chronic CGRP expression in TBI-affected brains. This pattern was also linked to an augmentation in cellular health within the ipsilateral hippocampal subfield. Aside from its impact on memory, TBI has been associated with functional outcomes related to mood. Prior investigations have suggested that CGRP is implicated in anxiety- and depressive-like behaviors [94, 95]. Nonetheless, the specific involvement of CGRP levels in behaviors subsequent to TBI requires elucidation for both male and female rats. Our findings indicate that elevated CGRP levels in the amygdala and thalamus are associated with ameliorated anxiety- and depression-like symptoms in females. This observation aligns with previous evidence supporting our results. Estrogen replacement therapy has demonstrated anxiolytic effects in ovariectomized rats [96–98]. Furthermore, the absence of estrogen has been associated with reduced spine and synaptic plasticity in the frontal cortex, resulting in cognitive and psychological impairments, such as dysfunction in contextual fear memory [96, 99–101]. Considering that the administration of CGRP for seven days post-TBI has been shown to alleviate TBI-induced anxiety [24], and its administration to both CGRP-sensitive and -insensitive mice reduces depressive behavior [95, 102, 103], it is plausible that the observed reduction in anxiety and depression in females may be mediated by the increase in CGRP expression. The contradictory effects of CGRP on anxiety and depression necessitate further investigation, taking into consideration the varying degrees of brain injury severity and distinct phases of traumatic brain injury TBI in both sexes.

There are limitations that should be considered. In the experiments reported here, the estrous cycle stage was not monitored in female rats, as our primary aim was to assess the potential identification of sexually dimorphic CGRP levels in TBI outcomes among a cohort of freely cycling females, in comparison to males. And indeed, we identified robust sex differences in CGRP levels without regard to hormonal conditions. However, it is possible that some sex differences are only present during certain stages of the estrous cycle and were thus masked in a group of females selected from randomly cycling estrous stages. The possibility that hormonal conditions at baseline, during the time of TBI, during the memory, anxiety- and depression-like behavior assessment, and during brain sample collections influence the CGRP response in different ways should be taken into account in future studies via staging or ovariectomy.

### Perspectives and significance

Our investigation unveiled significant sex disparities in CGRP expression within TBI brains. In light of recognized variations in TBI outcomes between males and females, coupled with documented sex-related distinctions in CGRP expression, it is imperative to systematically analyze the diverse mechanisms involving CGRP in TBI. Conducting such an inquiry is essential for attaining a thorough comprehension of the sex-specific functions exerted by CGRP throughout both the acute and chronic phases of TBI. Further, additional inquiries are necessary for identification of any negative interactions due to anti-CGRP monoclonal antibodies taken for the purpose of migraine prevention. Given the results presented in this manuscript, individuals taking anti-CGRP monoclonal antibodies would be liable to develop more severe damage following TBI. Following the determination of the therapeutic threshold of CGRP level in the injured brain across different severities, CGRP may represent a novel intervention with expansive therapeutic potential to be targeted in the context of TBI.

## Conclusions

Overall, the present findings reveal novel sex disparities in CGRP expression that influence both structural and functional outcomes subsequent to severe TBI. Our data further confirm that CGRP initiates Nrf2 and eNOS signaling pathways, leading to the mitigation of oxidative stress and enhancement of microcirculation following TBI, with a notable predominance in females. Females showed significantly higher CGRP levels which are associated with reduction in oxidative stress, microvascular dysfunction, white matter injury, and hippocampal injury, leading to improved memory function and reduced anxiety- and depression-like symptoms. Inhibition of CGRP resulted in exacerbation of oxidative stress, and decreased Nrf2 and eNOS expression in both males and females, with a more dominant response in females. This strongly suggests that CGRP may be one of key downstream mediators behind previously observed sexually dimorphic outcomes in TBI. While the precise mechanism and influencing factors governing the modulation of CGRP signaling in TBI brains are not yet fully understood, our results suggest that the activation of CGRP signaling correlates with enhanced TBI outcomes, as evidenced in female TBI brains.

## Data Availability

The datasets used and/or analyzed during the current study are available from the corresponding author on reasonable request.
